# Unravelling mechanisms that govern meiotic crossover formation in wheat

**DOI:** 10.1042/BST20220405

**Published:** 2022-07-28

**Authors:** James D. Higgins, Kim Osman, Stuart D. Desjardins, Ian R. Henderson, Keith J. Edwards, F. Chris H. Franklin

**Affiliations:** 1 Department of Genetics and Genome Biology, University of Leicester, Adrian Building, University Road, Leicester LE1 7RH, U.K.; 2 School of Biosciences, University of Birmingham, Birmingham B15 2TT, U.K.; 3 Department of Plant Sciences, University of Cambridge, Downing Street, Cambridge CB2 3EA, U.K.; 4 Life Sciences Building, University of Bristol, 24 Tyndall Avenue, Bristol BS8 1TQ, U.K.

**Keywords:** crossover, meiosis, recombination, wheat

## Abstract

Wheat is a major cereal crop that possesses a large allopolyploid genome formed through hybridisation of tetraploid and diploid progenitors. During meiosis, crossovers (COs) are constrained in number to 1–3 per chromosome pair that are predominantly located towards the chromosome ends. This reduces the probability of advantageous traits recombining onto the same chromosome, thus limiting breeding. Therefore, understanding the underlying factors controlling meiotic recombination may provide strategies to unlock the genetic potential in wheat. In this mini-review, we will discuss the factors associated with restricted CO formation in wheat, such as timing of meiotic events, chromatin organisation, pre-meiotic DNA replication and dosage of CO genes, as a means to modulate recombination.

## Introduction

Bread wheat (*Triticum aestivum*) is an allohexaploid (AABBDD, 2n = 6x = 42) that arose by hybridisation between tetraploid (*Triticum turgidum*; AABB, 2n = 4x = 28) and diploid *Aegilops tauschii* (DD, 2n = 2x = 14) in the fertile crescent of the Near East ∼10 000 years ago [1]. The *Triticum aestivum* genome is 14.07 Gb in size, comprised of subgenomes A (4.94 Gb), B (5.18 Gb) and D (3.95 Gb) [2–4]. Genetic analysis of chromosome 3B revealed an average of 2.6 crossovers (CO) per chromosome, and all COs observed were located in 13% of the chromosome [5]. Chromosomal regions exhibiting COs were located distally (to the centromere) in 68 Megabases (Mb) of the short arm and 59 Mb of the long arm (of 886 Mb total chromosome length), thus demonstrating a highly skewed bias towards the chromosome ends [5]. In addition, twenty-two CO hotspots were detected within these regions that possessed a recombination rate 10x the average, indicating local regions that were highly amenable for CO formation [5]. A CO hotspot at the glycosyl transferase gene *Hga3*A had previously been identified in wheat, indicating factors influencing CO formation at fine scales [6]. A global analysis of CO frequency in hexaploid wheat utilising molecular markers on 13 populations revealed a range of 41 to 52 COs per meiosis [7], consistent with 48 COs previously reported [8]. CO numbers in wheat have also been analysed cytologically through chiasma counts at metaphase I (MI) based on chromosome configurations, as well as quantifying foci of recombination proteins that mark COs during meiotic prophase I. For example, an average of 39 HEI10 foci per cell, which mark interference-sensitive class I CO sites were observed during pachytene/diplotene and 42 chiasmata at MI [9]. However, both cytogenetic measures are likely to be an underestimation of the total number of COs, as it is difficult to differentiate two closely spaced, adjacent chiasmata and HEI10-marked class I COs only account for 85% of the total number [9,10]. Chiasmata range from 34 to 48 per meiocyte in hexaploid wheat, indicating endogenous biological variability between cells and the majority (88%) were classified as distal, consistent with a previous molecular marker analysis [5,9]. Interestingly, the CO frequency for wheat chromosome 3B (2–3 per chromosome pair/meiosis) is comparable to the CO frequency on the longest *Arabidopsis thaliana* chromosome, even though there is a 32-fold size difference [11]. This indicates that constraints on CO formation appear substantially greater on the large wheat chromosomes in limiting COs to 1–3 per chromosome. In this review, factors governing wheat CO frequency and distribution will be discussed, as well as potential strategies to overcome such apparent constraints.

## Is the recombination pathway restricted in wheat?

In Arabidopsis, meiotic recombination is initiated by the formation of multiple programmed DNA double-strand breaks (DSBs) catalysed by the SPO11-1 [12] and SPO11-2 [13] heterodimer together with break-complex partners proteins MTOPVIB [14], PRD1-3 [15,16] and DFO [17]. In Arabidopsis, DSBs are more likely to form at gene promoters and terminators where the local chromatin structure is characterised by low nucleosome occupancy, low DNA methylation and adjacent enrichment of euchromatic marks histone 3 lysine 4 tri-methylation (H3K4me3) and the histone variant H2A.Z [18]. This chromatin environment may provide recombination proteins with greater accessibility to chromosomal DNA than regions of high DNA methylation and heterochromatin [18]. In maize, DSBs occur in all chromosomal regions including centromeres and peri-centromeres [19]. In contrast with Arabidopsis, only one quarter of DSBs form near genes in maize, whereas DSBs also form in repetitive DNA such as *Gypsy* retrotransposons [19]. However, in maize, only genic DSBs contribute to CO formation, so the purpose of DSB formation in the repetitive DNA remains unknown [19]. In wheat, fine-scale mapping of COs revealed an association with a DNA motif specific to the *TIR-Mariner* transposon family [20]. In addition, chromatin immunoprecipitation and sequencing (ChIP-seq) of the strand-exchange protein DMC1 (as a proxy for DSB formation) showed significant enrichment close to genes, and an overlap with transposable elements from the *Mariner* and *Mutator* families [21]. However, DMC1 hotspots found in CO-active regions were also strongly enriched for the Polycomb histone mark H3K27me3 [21]. H3K27me3 is distally enriched on the chromosomes and may play a role in promoting COs in wheat [21]. Therefore, it is likely that the local chromatin environment influences the potential for individual DSB sites in maturing into COs, as well as preventing ectopic recombination in repetitive elements that may cause genome instability [22].

In eukaryotes, the histone variant H2A.X becomes phosphorylated at DSB sites (to form γH2A.X) and therefore can be used as a marker to detect unrepaired DSBs in cytological preparations [23]. In wheat, ∼2100 evenly spaced γH2A.X foci co-localised with hyper-abundant sites of the chromosome axis protein ASY1 throughout the nucleus at leptotene, consistent with DSBs exhibiting interference, but also they are not limiting in number or position to potentially form COs [7]. As inter-chromosome sizes are similar in wheat, ∼2100 DSBs distributed across 21 chromosome pairs indicates that ∼100 DSBs lead to 1–3 COs per chromosome, and that 97–98% of DSBs are repaired as non-COs, either by repair from the homologue or sister-chromatid. It is not unusual for meiotic DSBs to be far in excess of COs in plants, as ∼200 DSBs lead to ∼10 COs in Arabidopsis [18], although the conversion rate of 5% in Arabidopsis is double that of wheat 2–3%. This raises questions including how many of these DSBs can potentially form a CO, and what is the probability of each DSB becoming a CO? It will be interesting to explore if DSBs can be modelled for their potential in becoming COs, by incorporating variables such as local chromatin environment. Further questions include, what is the upper limit for DSBs in forming COs per meiosis, and how many DSBs are required for correct chromosome pairing and synaptonemal complex formation? One interesting possibility is that wheat has evolved a weaker inter-homologue bias in order to reduce inappropriate interactions between homoeologous chromosomes. Current known factors limiting the conversion of DSBs into COs in wheat will be discussed in the following sections.

## Asynchronous timing of axis maturation and synaptonemal complex formation

Telomeres cluster into the ‘bouquet’ during early prophase I of multiple species, including wheat [9,24]. The role of the bouquet is proposed to bring chromosome ends into close proximity thereby promoting chromosome pairing and synapsis [24]. Early chromosome interactions are likely to bias the fate of DSBs towards CO formation, as observed in barley, a member of the Poaceae family that separated from wheat ∼13 million years ago [1,25,26]. However, centromeres also cluster in wheat during leptotene into the ‘Rabl’ formation [27], yet these interactions are less likely to promote the formation of COs from DSBs. This suggests that other factors are involved in limiting COs from forming in the centromeric/peri-centromeric regions.

**Figure 1.**
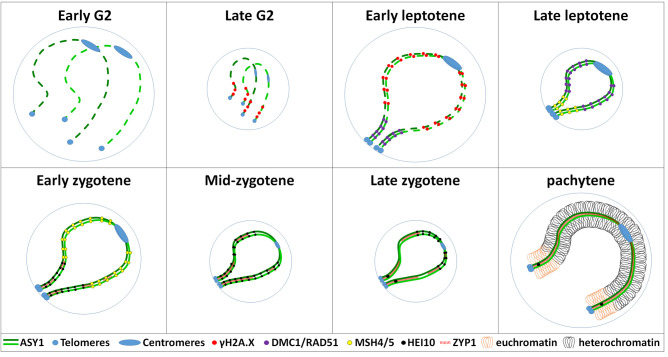
An illustration of meiotic prophase I progression in wheat highlighting factors that influence the distal bias of CO distribution and frequency. One stylised pair of wheat chromosomes is represented within the nuclear envelope in which the coordination of telomere/centromere clustering, chromosome axis maturation, chromosome pairing, synapsis and homologous recombination occur in the context of chromatin cycles of expansion and contraction. By Early G2, chromosomes have been replicated and are now a pair of sister-chromatids that are held together by the cohesion complex of proteins that form the axis where ASY1 begins to load. At late G2, the ASY1 protein becomes linear towards the telomeres and double-strand breaks are detected by γH2A.X labelling. During early leptotene, strand exchange proteins RAD51 and DMC1 localise to the linear ASY1 axis towards the telomeres and γH2A.X labelling is detected in the interstitial and centromere proximal regions. At this stage the telomeres have clustered into the bouquet and are located at the nuclear envelope. At late leptotene, class I CO proteins MSH4 and MSH5 are first observed co-localising with ASY1 adjacent to the telomeres and DMC1 and RAD51 are present in the interstitial/peri-centromeric regions. At this stage the chromosomes are fully paired. Early zygotene is characterised by the installation of the synaptonemal complex protein ZYP1 in the sub-telomeric regions and depletion of ASY1 as well as co-localisation of class I CO protein HEI10. MSH4 and MSH5 are observed in the interstitial/peri-centromeric regions. At mid-zygotene, ZYP1 has polymerised ∼50% of the chromosome pairs and HEI10 is observed in the majority of ZYP1/ASY1 labelled sites. At late zygotene, ZYP1 polymerisation is <75% and small HEI10 pairs of foci coalesce into singular, larger foci. At pachytene, ZYP1 polymerisation is complete and ASY1 is mostly depleted from the chromosome axis. HEI10 marks designated CO sites that form predominantly in the distal regions where the euchromatin is hyper-abundant. All prophase I chromosomes contain loops with euchromatin and heterochromatin abundant regions, but for ease of presentation these are only shown at pachytene.

The asynchronous loading of the chromosome axis protein ASY1 [28] and the synaptonemal complex transverse filament protein ZYP1 [29] are likely to be instrumental. ASY1 localises to meiotic chromosomes from G2 to diplotene, promoting inter-homologue recombination, thereby enabling accurate pairing, synapsis and recombination [30]. In wheat, ASY1 first appears as foci during G2 that extend into short stretches, coincident with telomere clustering [9] (Figure 1). However, within the chromosomes, this process is asynchronous, as when mature axes form adjacent to the telomeres the centromeric/interstitial regions still exhibit ASY1 foci [9] (Figure 1). Wheat *ASY1*^RNAi^ lines exhibited multivalents and univalents, suggesting that a reduced dosage of *ASY1* is insufficient to maintain accurate inter-homologue recombination and CO formation [31], although this may be due to an indirect effect by a delay of meiotic progression. ChIP-seq of ASY1 during prophase I in wheat revealed hyper-abundance at the distal ends of the chromosomes that may reflect the asynchronous timing or that there is simply more ASY1 protein located in the distal regions [21]. In contrast, ASY1 in Arabidopsis is hyper-abundant in the chromosomal interstitial regions, consistent with a significant effect on genome architecture [32]. It follows that localisation of wheat ASY1 would therefore consolidate the sub-telomeric bias for CO formation in wheat.

The synaptonemal complex transverse filament protein ZYP1 physically tethers the chromosome axes together during zygotene/pachytene, ensuring the formation of the obligate CO (every chromosome pair receives at least one CO) and CO interference (the phenomenon that reduces the probability of two closely spaced COs) [33–35]. As the ASY1 signal becomes linear throughout the nucleus during leptotene, ZYP1 loading at synapsis initiation sites commences in the distal regions (Figure 1). However, it is worth noting that ZYP1 is observed as numerous foci throughout the nucleus during lepotene, but it is unclear whether this represents protein aggregates that are localising specifically to the chromosomes, or pools of protein that are in preparation for chromosome synapsis [9]. Short stretches of ZYP1 are first observed in the sub-telomeric regions that then extend during zygotene as further synapsis initiation sites are detected throughout the nucleus [9] (Figure 1). Recent studies from Arabidopsis have revealed that ZYP1 is responsible for limiting closely spaced COs [34,35] and therefore, ZYP1 polymerising from the chromosome ends could be a mechanism that prevents COs from forming in the centromeric and interstitial regions. However, barley *ZYP1^RNAi^* lines exhibit a significant reduction in chiasma, including loss of the obligate chiasma, indicating that ZYP1 promotes formation of class I COs [33]. The asymmetric loading of ASY1 and ZYP1 in wheat correlates with the asynchronous timing of meiotic progression as determined by a BromodeoxyUridine (BrdU) time-course [9]. This pattern of meiotic progression has previously also been reported in diploid barley [25], indicating that it is independent of polyploidisation and most likely arose in the progenitor diploid grasses, or earlier. The recombination proteins DMC1, RAD51, MSH4, MSH5 and HEI10 are also subject to the asynchronous loading during meiotic prophase I, and this pattern is likely to be co-ordinated by the chromosome axis and synaptonemal complex proteins [9] (Figure 1).

## Chromatin organisation correlates with crossover formation

Gene density is highest at the distal ends of the chromosomes, correlating with an enrichment of euchromatin marks H3K27me3, H3K4me3, H3K9ac and H3K36me3 [2,36]. Specifically, the Polycomb histone modification H3K27me3 is enriched at ‘young’ non-conserved genes in the distal regions that experience a high CO rate, compared with ‘old’ genes that are highly similar in hexaploid wheat to the diploid and tetraploid progenitors [36]. DMC1 and ASY1 enrichment observed by ChIP-seq and immunocytology also correlates with H3K27me3, indicating a role for facultative heterochromatin in CO bias, in addition to gene density and transposable elements in the *Marine*r and *Mutator* superfamilies [21]. DNA methylation and the heterochromatin mark H3K9me2 are substantially depleted in the distal chromosomal regions, compared with interstitial and centromeric regions, suggesting that ‘open’ euchromatin influences CO formation in wheat [36]. However, as CO formation and chromatin conformation correlate with each other, the association may be direct or indirect. For example, in barley, distal euchromatin-enriched regions were first to replicate during pre-meiotic S-phase and therefore enter meiosis ahead of the late-replicating interstitial and proximal regions. This indirect temporal advantage appears to be sufficient to bias CO designation to the distal regions [25] and this pattern has also been observed in wheat [9].

The timing of meiotic progression is co-ordinated with cycles of chromatin expansion and contraction during prophase I in barley and wheat [9,25] (Figure 1). It is postulated that this is a manifestation of the mechanical model of CO interference [37], whereby physical forces generated by the chromosome loops that are attached to the axes drive key events in the meiotic program. Therefore, if distal recombination events are synchronised with the chromatin cycles, but the interstitial/proximal regions are not, then the CO bias remains. Synchronising timing events along the chromosomes may be required to reduce the distal bias for CO formation. Barley grown at 30°C may have increased the accessibility of the chromatin in the interstitial regions to replication factors, thereby partially synchronising the distal and interstitial regions entering meiosis, leading to an increased frequency of interstitial chiasmata [25]. In wheat as in barley, key meiotic events occur when the chromosome loops in the nuclei are expanded and would exert the most mechanical force on the chromosome axes [9].

## Altered gene dosage of class I crossover genes

The class I CO pathway in wheat is responsible for 85% of COs, as well as maintaining the formation of the obligate CO, so that every pair receives at least one CO event that is essential for accurate chromosome segregation [10]. In addition, the class I CO pathway appears more tolerant of recombining in polymorphic chromosome regions that are heterozygous compared with the class II CO pathway in Arabidopsis [38–40] and therefore more likely to form COs between homoeologous chromosomes in wheat. The *Pairing homoeologous* (*Ph1*) locus was identified over 60 years ago as responsible for controlling the diploid-like behaviour of chromosome pairing in hexaploid wheat [41]. The *Ph1* locus has now been genetically mapped, which has revealed that an additional, highly expressed copy of the class I CO gene *ZIP4* on chromosome 5B is responsible for promoting homologous over homoeologous recombination in wheat [42]. A null *ZIP4-5B* mutant recapitulated the *ph1b* deletion phenotype and recent analysis has shown that there are two domains in TaZIP4-B2 protein, one controlling pairing and synapsis between homologues, the other suppressing COs between homoeologues [43]. The role of the *Ph1 ZIP4* gene copy is not fully understood, but in budding yeast the orthologous protein couples CO formation to synaptonemal complex assembly [44]. In rice, ZIP4 interacts with the MutSγ heterodimer MSH4/MSH5 and is required for normal levels of COs [10,45]. In tetraploid and hexaploid wheat, the *MSH5B* gene is naturally mutated, and in hexaploid wheat the *MSH4D* gene has been naturally mutated, thus reducing the gene dosage of MutSγ [10]. Loss of gene copies is a common event in redundant polyploid genomes by fractionation, but meiotic recombination genes return to a single copy more rapidly than the genome average in species analysed [46]. In polyploid *Brassica napus*, reducing the copy number of *MSH4* prevented COs from forming between non-homologous chromosomes [47]. Therefore, the natural mutation of *MSH5B* and *MSH4D* in wheat may represent an adaptation to prevent homoeologous recombination, although this hypothesis needs functional testing. Reduced gene dosage of MutSγ may also limit stabilisation of recombination complexes between homologues, thus limiting CO formation. In Arabidopsis, overexpression of class I CO protein HEI10 increased recombination [48]. Overexpressing HEI10 in wheat may have the desired effect of increasing recombination, although three functional *HEI10* gene copies may be sufficient to produce enough protein in the wild type that increasing levels has no effect. In addition, naturally altered gene dosage of *ZIP4* and *MutSγ* should be taken into consideration if overexpressing HEI10, or other members of this pathway as they may be rate limiting.

## How do DNA repair genes modulate recombination?

The positional cloning of the *Pairing homoeologous 2* (*Ph2*) locus and functional validation revealed that the underlying gene encodes the mismatch repair protein MSH7-3D [49]. Mutating *MSH7-3D* increased homoeologous recombination up to 5.5-fold in wheat-wild relative hybrids, suggesting that it acts to prevent COs from forming between divergent DNA sequences [49]. *MSH7-3A/3B* are still active in *ph2* mutants, implying that gene dosage is critical in modulating outcomes in the wheat recombination pathway. In Arabidopsis, MSH2 forms a heterodimer with MSH7 and promotes the formation of COs at polymorphic DNA sites [50], thus appearing to function differently to wheat [40]. The role of mismatch repair proteins during meiosis in plants is incompletely understood and they may act at multiple steps of the recombination pathway in detecting heteroduplex DNA and consequently promoting or rejecting progression of intermediates from forming a CO.

The anti-recombinases FANCM, FIGL-1 and RECQ4 have been identified in Arabidopsis [51–54], but not fully characterised in wheat. A VIGS approach has been utilised to analyse the effect of *FANCM* hypomorphs on recombination in F_1_ tetraploid wheat hybrids, where silencing had no effect on CO numbers, but altered CO distributions [55]. We have recently shown that FANCM promotes formation of class I COs, whilst preventing the formation of class II COs in tetraploid and hexaploid wheat [56]. In tetraploid wheat, the gain in class II COs is equal to the loss of class I COs, thus no net change in recombination occurred in *fancm*. However, in the hexaploid *fancm* null mutant, the loss of class I COs is relatively less than the tetraploid, giving an overall 31% increase in COs. This gain in COs associates with H3K27me3 and the additional COs are likely to be in the same ‘hot’ regions as wild type [56].

The DNA helicase RECQ7 is a promising candidate for modulating recombination in wheat [7]. RECQ7 was identified as a QTL associating with increased CO formation and gene conversion in wheat recombinant inbred lines [7]. The recombination role of RECQ7 was validated with TILLING mutant lines that experienced a decrease in the frequency of gene conversions and COs compared with wild type [7]. Gene conversions form at a much higher frequency than COs and are not limited to distal chromosomal locations in wheat, thus providing an alternative mechanism for transferring information from one chromosome to another in recombinationally ‘cold’ regions [7].

## Perspectives

- CO formation is limited in number and position to the chromosome ends in wheat, thus restricting the potential for exchange of regions with advantageous agronomical traits located in the centromere-proximal and interstitial regions. Therefore, understanding the factors influencing CO formation may enable routes for expediting the improvement of wheat varieties via breeding approaches.
- Wheat chromosomes are characterised by H3K27me3 enriched regions towards the chromosome ends that strongly positively correlate with CO number and position [21].
- Gene dosage of recombination genes appears to be a key adaptation for successful meiotic recombination as well as timing of chromosome axis and synaptonemal complex formation.
- Modulating meiotic genes controlling recombination, chromosome axis elaboration/synaptonemal complex formation and chromatin structure may provide suitable targets for increasing recombination in ‘cold’ regions.

## Open Access

Open access for this article was enabled by the participation of University of Birmingham in an all-inclusive *Read & Publish* agreement with Portland Press and the Biochemical Society under a transformative agreement with JISC.

## Abbreviations

CO: crossovers
DSBs: double-strand breaks
MI: metaphase I
